# Material Supplies for Our Profession

**Published:** 1871-11

**Authors:** 


### MATERIAL SUPPLIES FOR OUR PROFESSION.

When we remember the manner in which our profession was supplied with all instruments, appliances and materials, twenty-five years ago, and now contemplate the manner in which it is done, it affords matter of wonder and astonishment.

Thoughts like this recently ran in rapid succession through our mind, while making, or endeavoring to make an examination of S. S. White's Dental Depot, in Philadelphia. He has as a beginning, in many respects, the finest building we ever saw, completely fireproof, and of the most substantial character throughout.

The establishment consists of numerous departments, each with its battalion of workmen, controlled and directed by a superintendent, and all these by one director-general. Every department is supplied with all the facilities, that the most extended acquaintance, and familiarity with its requirements can devise, and the most lavish expenditure of money procure.

In all these departments, the most persevering and extended course of experiments have been conducted, with a view of attaining the highest results, in the procurement of instruments and materials for the dentist's use.

In one department alone within the last three years, over forty thousand dollars have been expended in experimenting with steel. Many of the processes employed in this establishment have no equal in this country, neither in the manner of accomplishment nor in the results obtained. Here are talent, genius, and capital combined in an eminent degree, and co-operating in the production of that which shall be most efficient in mitigating or relieving some of the greatest afflictions to which our fellow men are subject. T.

### TO THE PROFESSION.

At the last meeting of the American Dental Association, a Committee on Local Societies and Conference with the Profession was appointed. The following persons constitute that committee: Prof. H. Judd, St. Louis, Mo.; Prof. J. Taft, Cincinnati, O.; Prof. H. W. Atkinson, New York City; Prof. L. D. Shepard, Boston, Mass.; Dr. Wm. Dutch, San Francisco, Cal. The object of this committee is to aid and encourage the organization and efficient operation of local societies, hold clinics, etc., wherever and whenever opportunity may offer. T.

